# Comparison of Intravenous Ketamine with Intrathecal Meperidine in Prevention of Post-anesthetic Shivering after Spinal Anesthesia for Lower Limb Orthopedic Surgeries: A Double-blind Randomized Clinical Trial

**DOI:** 10.4314/ejhs.v31i6.16

**Published:** 2021-11

**Authors:** Abdolmajid Gholinataj, Afshin Gholipour Baradari, Soheila Najafi, Farshad Hasanzadeh Kiabi

**Affiliations:** 1 Department of Anesthesiology, Faculty of Medicine, Mazandaran University of Medical Sciences, Sari, Iran

**Keywords:** Shivering, Postoperative Shivering, Meperidine, Spinal Anesthesia, Randomized Clinical Trial

## Abstract

**Background:**

Post-anesthetic shivering is one of the most common complications after anesthesia. Ketamine has been considered to be an effective treatment for post-anesthetic shivering, but the evidence for its therapeutic benefit after spinal anesthesia is limited. The aim of this study was to compare the effects of intravenous ketamine with intrathecal meperidine in the prevention of post-anesthetic shivering after spinal anesthesia for lower limb orthopedic surgeries.

**Methods:**

In a double-blind randomized parallel-group clinical trial, a total of 150 patients scheduled for lower limb orthopedic surgeries under spinal anesthesia were selected and randomly divided into three equally sized groups of intravenous ketamine (0.5 mg/kg), intrathecal meperidine (0.2mg/kg) or intravenous normal saline (as placebo). The intensity of shivering in patients were evaluated during surgery and after transfer into the post anesthesia care unit. Also, changes in patients' drowsiness, nausea, vomiting, pruritus, mean arterial pressure, heart rate, and arterial oxygen saturation (SPO2) during surgery and until the end of anesthesia were evaluated.

**Results:**

In all times of evaluation (20, 60, 80, 100 and 120 minutes after onset of spinal anesthesia) patients in control group showed a greater intensity of shivering compared to other groups. However, patients who received intrathecal meperidine experienced significantly lower intensity of post anesthetic shivering (p<0.05). The results showed a significant mean arterial pressure and heart rates differences between the three groups, only on 20 and 60 minutes after initiation of spinal anesthesia. The incidence of nausea, vomiting, and pruritus was not significantly different in all three groups, although all patients who received ketamine experienced drowsiness after surgery (p<0.001).

**Conclusion:**

The results of the present study showed that, although both intrathecal meperidine and intravenous ketamine could effectively prevent postoperative shivering after spinal anesthesia in lower limb orthopedic surgeries, intrathecal meperidine was associated with more efficacy benefits and a lower frequency of side effects such as post-anesthesia drowsiness.

## Introduction

Postoperative shivering is one of the most common complications after general or regional anesthesia as an unpleasant and stressful experience in patients, which can exacerbate postoperative pain (1). The prevalence of postoperative shivering has been reported in various studies between 40% and 70% (2, 3). Normally, despite changes in the outside temperature, the autonomic nervous system maintains the original temperature between 36.5 °C and 37.5 °C by making behavioral and physiological changes (4, 5). During anesthesia, thermoregulation is usually impaired. Also, patients are usually exposed to cool environment in the operating room. On the other hand, the use of cold liquids exacerbates hypothermia (5, 6). Persistent hypothermia can lead to complications such as postoperative shivering, coagulopathy, decreased immunity, cardiac morbidity (7), postsurgical wound infection, impaired wound healing, prolonged recovery from anesthesia and prolonged hospital stay (6, 8, 9).

Prevention or treatment of postoperative hypothermia and shivering consists of pharmacological and non-pharmacological interventions. Non-pharmacological modalities include the use of heating blankets, keeping the patient warm and preventing temperature loss before and after surgery, inhaling hot and humid oxygen, and preventing the over-cooling of the operating room environment. Despite the promising results, the efficacy of these nonpharmacological treatments are largely limited and unknown (6, 10). Various pharmacological agents such as meperidine, Ondansetron, alfentanil, clonidine, and magnesium sulfate are also used to treat postoperative shivering (1). Although these drugs are effective in reducing postoperative shivering with various mechanisms, they may be problematic in terms of side effects such as loss of consciousness, respiratory depression, pruritus, and nausea and vomiting (11).

Although different strategies are used for postoperative shivering management, there is no proven strategy to prevent it. Among opioids, meperidine is known for its potential ability to treat postoperative shivering after general and regional anesthesia. In addition, it has been shown that the effect of meperidine on postoperative shivering control is better than morphine and fentanyl (12). Ketamine, as a competitive N-methyl-D-aspartic acid (NMDA) receptor antagonist, is another potential agent of interest for postoperative shivering control (13). It is believed that using drugs in combination may be more efficacious than single-drug therapy for treatment of postoperative shivering. The results of a study comparing the effects of using intravenous ketamine, midazolam and the combination of ketamine and midazolam showed that patients who received the combination drug therapy experienced significantly lower rate of postoperative shivering (14). Although some studies indicate the efficacy of ketamine in prevention of postoperative shivering (12, 13), these results were not confirmed by other studies (15).

Considering the potential preventive efficacy of combination of intravenous ketamine with intrathecal meperidine for postoperative shivering, the aim of this study was to compare the effects of intravenous ketamine with intrathecal meperidine in the prevention of postanesthetic shivering after spinal anesthesia for lower limb orthopedic surgeries.

## Methods

**Study design and sample**: In a double-blind randomized parallel-group clinical trial, a total of 150 patients who were admitted to Imam Khomeini Hospital in Sari, Iran, for lower limb orthopedic surgery using spinal anesthesia were evaluated. The study was conducted between March 2017 and January 2019.

**Inclusion and exclusion criteria**: The inclusion criteria were elective lower limb orthopedic surgery (knee and below-knee), using spinal anesthesia, age between 18–65 years, ASA Class I, II, no drug addiction, no neurological diseases, no fever, no symptoms of high intracranial pressure such as headache, nausea, vomiting, blurred vision, diplopia), BMI<30, no contraindications for spinal anesthesia known and allergy to meperidine or ketamine. Patient dissatisfaction with continuing to participate in the study, preoperative temperature >38°C or <35°C, receiving blood or blood products during surgery and occurrence of any complications during surgery were considered as exclusion criteria.

**Intervention**: Eligible patients were randomly allocated into three groups (two intervention groups and one control group), with an equal sample size. In the first group of interventions, 15 mg of bupivacaine 0.5% in normal saline (total volume of 4 ml) was administered intrathecally. Immediately after spinal anesthesia, 0.5 mg/kg of ketamine in normal saline (total volume of 5 ml) was intravenously infused. In the second group of interventions, 15 mg of bupivacaine 0.5% and 0.2 mg/kg of meperidine in normal saline (total volume: 4 ml) was injected intrathecally. In addition, immediately after spinal anesthesia five ml of normal saline was infused intravenously. In the control group, 15 mg of bupivacaine 0.5% in normal saline was injected intrathecally (total volume of 4 ml). Also, five ml of normal saline was infused intravenously. During the present study, the intraoperative room temperature was maintained at 23–25°C. Also, all patients received intravenous fluids at room temperature.

**Randomization and blinding:** Eligible patients were divided into three equal groups to participate in the study, using a computerized list of random numbers. Patients were blinded with regard to study groups. Also, a nurse anesthetist who were unaware of patients' group, assessed the outcomes of the study. To ensure allocation concealment, the allocation sequence was constructed by an anesthesiologist who was not involved in recruitment.

**Data collection**: In this study, a researcher-made checklist was used to collect data. This checklist was included patients' demographic and clinical characteristics such as age, sex, weight, height, mean arterial systolic and diastolic blood pressure, heart rate, oxygen saturation (SPO2), pruritus, nausea, vomiting, drowsiness-restlessness and the duration of spinal anesthesia and surgery. These data were recorded immediately before the start of anesthesia and every 20 minutes until the end of the surgery and admission to the post anesthesia care unit. The checklist was completed by a nurse who was blinded from the study groups and had received sufficient training to fill the questionnaire. In addition, at the beginning of the study, patients' weight was measured in kg and height in cm, and the BMI of the patients was calculated by dividing the weight (kg) by the square height (m^2^).

**Outcomes**: In this study, the primary outcome was the intensity of shivering which was evaluated at 3 minutes before the onset of spinal anesthesia, as well as 20, 60, 80, 100 and 120 minutes after the onset of spinal anesthesia. Shivering was graded with a defined scale by Crossley et al. (16); as 0, no shivering; 1, piloerection or peripheral vasoconstriction with no visible shivering; 2, muscular activity in one muscle group; 3, muscular activity in more than one muscle group, but not generalized shivering; and 4, shivering involving the whole body.

The secondary outcomes were the comparison of changes in patients' drowsiness-restlessness according to the Richmond agitation-sedation scale, nausea, vomiting, pruritus, arterial blood pressure, heart rate, and SPO2 during surgery until the end of anesthesia.

**Ethical consideration:** This study was performed in Imam Khomeini Hospital, Sari after receiving the approval of the ethics committee of Mazandaran University of Medical Sciences. After explaining the aim of the study, the researchers obtained informed consent from the participants. Also, the study was registered at the Iranian Registry of Clinical Trials Database (IRCT20160403027191N3).

**Sample size calculation**: A priori sample size was calculated using GPower3.1 with the formula for calculation of samples of repeated measures, based on a presumed effect size of 0.1, a statistical power of 80%, and a type I error of 5%. The overall proper sample size was found to be 135 participants. We therefore recruited 150 patients to account for any dropouts.

**Statistical analysis**: Data were analyzed using SPSS software package (version 16.0, SPSS Inc., Chicago, IL, USA). In this study, descriptive statistics (mean, standard deviation) and inferential statistics [Chi-square, T-test and repeated measures analysis of variance (ANOVA)] were used. P-value <0.05 was considered statistically significant.

**Data sharing**: All relevant data and methodological detail pertaining to this study are available to any interested researchers upon reasonable request to corresponding author.

## Results

In total, 150 patients were included in the study, which was divided into three groups with an equal sample size (Figure 1). In the intravenous ketamine, intrathecal meperidine and control groups 64%, 76%, and 72% of patients were male. The mean age of patients in the ketamine intravenous, intrathecal meperidine, and control groups was 41.24 (SD=12.71), 40.4 (SD=15.51), and 39.69 (SD=11.31) years, respectively. Other demographic and clinical characteristics of patients were shown in Table 1.

**Table 1:** Basic demographic characteristics of patients in three groups

Variable		Groups			*P-value*
			
		Intravenous ketamine (n=50)	Intrathecal meperidine (n=50)	Control (n=50)	
Age, year		41.24 (SD=12.71)	40.4 (SD=15.51)	39.69 (SD=11.31)	0.167
Sex	Male	32 (64)	38 (76)	36 (72)	0.064
	Female	18 (36)	12 (24)	14 (28)	
Weight (kg)		70.32	67.24	69.32	0.30
Duration of anesthesia (minutes)		95.23	100.04	90.12	051

Data are presented as mean ± standard deviation or number (percentage)

**Primary Outcome**

**Shivering**: Before onset the anesthesia, there was no differences between three groups in terms of intensity of shivering. However, there was a significant difference between three groups (intravenous ketamine, intrathecal meperidine, and control groups) in terms of shivering intensity after initiation of spinal anesthesia. So that in all times of evaluation (20, 60, 80, 100 and 120 minutes after onset of spinal anesthesia) patients in control group showed a greater intensity of shivering compared to other groups (p<0.05) (Table 2).

**Table 2:** Mean and standard deviation of Intensity of shivering and hypothermia in three groups

	Intensity of shivering				Hypothermia			
	
	Ketamine (n = 50)	Meperidine (n = 50)	Control (n = 50)	p- value	Ketamine (n = 50)	Meperidine (n = 50)	Control (n = 50)	p- value
3 minutes before the onset of spinal anesthesia	0.75 (SD=0.14)	0.63 (SD=0.38)	0.92(SD=0.58)	0.120	36.5 (SD=1.21)	36.7 (SD=2.8)	36.5 (SD=1.4)	0.142
20 minutes after the onset of spinal anesthesia	0.42 (SD=0.54)	0.34 (SD=0.03)	0.70(SD=0.05)	<0.001	36.9 (SD=1.3)	36.9 (SD=1.9)	36.1 (SD=0.2)	0.106
60 minutes after the onset of spinal anesthesia	1.90 (SD=0.36)	1.15 (SD=0.25)	3.22(SD=0.19)	<0.001	35.5 (SD=2.5)	36.2 (SD=2.6)	34.8(SD=2.12)	0.037
80 minutes after the onset of spinal anesthesia	1.21 (SD=0.23)	0.85 (SD=0.23)	2.10(SD=0.14)	0.048	35.8 (SD=2.1)	36.8 (SD=2.7)	35.0(SD=2.61)	0.035
100 minutes after the onset of spinal anesthesia	0.91 (SD=0.10)	0.33 (SD=0.02)	1.45(SD=0.06)	0.020	36.3 (SD=2.05)	37.0 (SD=2.59)	35.9(SD=2.43)	0.005
120 minutes after the onset of spinal anesthesia	0.52 (SD=0.03)	0.25 (SD=0.00)	0.85(SD=0.03)	0.014	36.5 (SD=2.4)	37.2 (SD=2.46)	36.3(SD=2.82)	0.015

**Secondary Outcomes**

**Hypothermia**: The results showed that before the onset of spinal anesthesia, there was no differences between three groups in terms of the occurrence of hypothermia (p=0.142). However, there was a significant difference between three groups in terms of hypothermia after 60, 80, 100 and 120 minutes after onset of spinal anesthesia. Patients in control group experienced greater degree of hypothermia in abovementioned time intervals compared to other groups (p<0.05) (Table 2).

**Changes of SPO2, MAP and Heart rate**: The results showed that there were no significant differences between three groups in terms of SPO2, in all times interval of evaluation.

Regarding MAP and heart rate, the results showed a significant MAP and heart rates differences between the 3 groups, only on 20 and 60 minutes after initiation of spinal anesthesia (Table 3).

**Table 3:** Mean and standard deviation of SPO2, MAP and heart rate in three groups

	Oxygen saturation (SPO2)				Mean arterial pressure (MAP)				Heart rate			
	
	Ketamine (n = 50)	Meperidine (n = 50)	Control (n = 50)	p- value	Ketamine (n = 50)	Meperidine (n = 50)	Control (n =50)	p- value	Ketamine (n = 50)	Meperidine (n = 50)	Control (n =50)	p- value
3 minutes before the onset of spinal anesthesia	98.2(SD=5.12)	98.3(SD=4.62)	98.5(SD=5.21)	0.087	109.15	100.21	98.56	0.230	79.41	78.85	78.65	0.213
20 minutes after the onset of spinal anesthesia	95.3(SD=4.32)	98.1 (SD=5.64)	98.5(SD=5.41)	0.540	100.32	80.23	80.02	0.019	90.75	70.64	65.52	0.013
60 minutes after the onset of spinal anesthesia	95.1(SD=5.31)	98.5 (SD=5.33)	99.2(SD=4.94)	0.141	95.26	90.32	90.35	0.047	85.63	70.12	70.24	0.027
80 minutes after the onset of spinal anesthesia	96.2(SD=5.22)	98.3 (SD=4.28)	98.3(SD=5.78)	0.850	95.68	95.27	90.74	0.480	80.74	75.46	75.26	0.420
100 minutes after the onset of spinal anesthesia	98.5(SD=4.84)	99.1 (SD=5.26)	99.5(SD=4.23)	0.221	105.38	103.45	95.27	0.540	81.45	80.85	80.41	0.510
120 minutes after the onset of spinal anesthesia	98.3(SD=4.61)	99.7 (SD=5.64)	99.2(SD=4.79)	0.412	105.68	103.55	101.37	0.120	80.56	79.89	79.23	0.142

**Side effects**: The results showed that the rate of vomiting in the intravenous ketamine group was 4% (2 cases in 50 patients), in the intrathecal meperidine group was 4% (2 cases in 50 patients), and the in the control group was 4% (2 cases in 50 patients) (p=0.323). Nausea was occurred in 4 cases (8%) of the intravenous ketamine group, 6% in the intrathecal meperidine group (3 cases), and 8% in the control group (4 cases) (p=0.132). None of patients in intravenous ketamine and control groups experienced pruritus after spinal anesthesia, however, 4% (2 cases) of patients in the intrathecal meperidine group had pruritus after spinal anesthesia (p=0.210). The rate of drowsiness in the intravenous ketamine group was 100%. However, post-anesthesia drowsiness was not occurred in any of patients in the intrathecal meperidine and control groups (p<0.001) (Table 4).

**Table 4:** Occurrence of side effects in three groups

Side effects		Groups			*P*-value
			
		Intravenous ketamine n (%)	Intrathecal meperidine n (%)	Control n (%)	
**Vomiting**	Yes	2 (4)	2 (4)	2 (4)	0.323
	No	48 (96)	48 (96)	48 (96)	
**Nausea**	Yes	4 (8)	3 (6)	4 (8)	0.132
	No	46 (92)	47 (94)	46 (92)	
**Pruritus**	Yes	0 (0)	2 (4)	0 (0)	0.210
	No	50 (100)	48 (96)	50 (100)	
**Drowsiness**	Yes	50 (100)	0 (0)	0 (0)	<0.001
	No	0 (0)	50 (100)	50 (100)	

Data are presented as number (percentage)

## Discussion

The results of the present study showed that, although both intrathecal meperidine and intravenous ketamine could effectively prevent postoperative shivering after spinal anesthesia in lower limb orthopedic surgeries, intrathecal meperidine was associated with more efficacy benefits and a lower frequency of side effects such as pruritus. In recent years, researchers have investigated the effects of various pharmacological agents for prevention or treatment of postoperative shivering (1, 17–18). Ketamine has been considered as a drug with the anesthetic function, which can change the temperature-regulating system and prevent postoperative shivering. In recent years, much attention has been paid to the efficacy of ketamine in chronic pain management, postoperative pain control and prevention of postoperative shivering (19). In line with the results of the present study, the results of a study in Turkey showed that there was no difference between meperidine and ketamine in prevention of postoperative shivering after general anesthesia (20). Also, in other studies the preventive efficacy of intravenous ketamine for postoperative shivering after spinal anesthesia has been confirmed (21, 22). The results of a study showed the prophylactic effect of ketamine for postoperative shivering after general anesthesia (23, 24). Another study confirmed the safety and prophylactic efficacy of epidural ketamine for prevention of shivering in patients undergoing caesarean section during combined spinal epidural anesthesia (25). In comparison of two doses of 0.5 and 0.3 mg/kg of intravenous ketamine, the results of a similar study in Iran showed that ketamine at a dose of 0.5 mg/kg has a significant effect on reducing postoperative shivering compared to the dose of 0.3 mg. However, meperidine had a more favorable effect on reducing postoperative shivering (26). The results of another study showed that regardless of dose (0.25 mg/kg or 0.5 mg/kg), intravenous ketamine significantly reduced shivering in patients following elective abdominal hysterectomy (27).

In our study it has been revealed that patients who received intravenous ketamine had higher MAP and HR compared to patients who received intrathecal meperidine. Although, these differences were statistically significant only in 20 and 60 minutes after anesthesia. This finding was not supported by other studies that evaluated the prophylactic effects of ketamine for postoperative surgery (28, 21). In contrast, another study revealed that there was no significant differences between using ketamine and meperidine in prevention of post anesthetic shivering (29). According to the results of the present study, there was no significant difference between the three groups in terms of side effects of drugs such as nausea, vomiting, and pruritus, but drowsiness was significantly higher in patients who received ketamine. This finding indicates a stronger effect of sedative ketamine at a dose of 0.5 mg/kg compared to lower doses as well as meperidine (20). Although most studies confirm the antishivering effect of ketamine, it has been reported that meperidine was a better and more effective choice for shivering prevention in most studies. Central nervous system and respiratory depression are the main problem with narcotics, such as meperidine. In particular, this complication may be exacerbated in patients who have undergone surgery and anesthesia and have previously received sedative and narcotic (30). However, the usual prescription of meperidine is more logical and safer (31). Overall, although our study showed that both ketamine and meperidine had antishivering effects, intrathecal meperidine had a better efficacy. One of the limitations of the present study was the inability to eliminate the surgical associated factors in post anesthetic shivering. In future studies, it is best to consider the surgical factors involved in shivering and to monitor the patient's temperature rectally during the operation, which was not possible in the present study. On the other hand, the single-center location of this study and the lack of control over room temperature and IV fluid are other limitations of this study. In conclusion, according to the results of present study, it seems that intrathecal meperidine can be used as an effective and low-risk pharmacologic intervention for prevention of post anesthetic shivering after spinal anesthesia of lower limb orthopedic surgery.
